# Predictors for distant metastasis in head and neck cancer, with emphasis on age

**DOI:** 10.1007/s00405-020-06118-0

**Published:** 2020-06-15

**Authors:** Martine F. van der Kamp, Friso O. W. Muntinghe, René S. Iepsma, Boudewijn E. C. Plaat, Bernard F. A. M. van der Laan, Ayat Algassab, Roel J. H. M. Steenbakkers, Max J. H. Witjes, Boukje A. C. van Dijk, Geertruida H. de Bock, Gyorgy B. Halmos

**Affiliations:** 1 Department of Otorhinolaryngology/Head and Neck Surgery, University of Groningen, University Medical Centre Groningen, P.O. Box 30.001, 9700 RB Groningen, The Netherlands; 2 Department of Radiation Oncology, University of Groningen, University Medical Centre Groningen, Groningen, The Netherlands; 3 Department of Oral and Maxillofacial Surgery, University of Groningen, University Medical Centre Groningen, Groningen, The Netherlands; 4 Department of Research and Development, Netherlands Comprehensive Cancer Organization (IKNL), Utrecht, The Netherlands; 5 Department of Epidemiology, University of Groningen, University Medical Centre Groningen, Groningen, The Netherlands

**Keywords:** Distant metastasis, Head and neck squamous cell carcinoma, Age, Hypopharyngeal cancer, Regional lymph node metastasis, Histological differentiation grade

## Abstract

**Purpose:**

Distant metastasis (DM) in patients with head and neck squamous cell carcinoma (HNSCC) is uncommon, but strongly deteriorates prognosis. Controversy exists regarding age as a predictor for the presence and development of DM. The aim of this study was to investigate age and other predictors for DM in HNSCC patients.

**Methods:**

From 1413 patients diagnosed with a primary HNSCC between 1999 and 2010 in a tertiary referral centre, patient, disease and pathological characteristics were extracted from patient files. Uni- and multivariable Cox regression analyses were performed to identify risk factors for DM as primary outcome.

**Results:**

DM occurred in 131 (9.3%) patients, of which 27 (1.9%) were diagnosed simultaneously with the primary tumour, 27 (1.9%) were diagnosed synchronous, and 77 (5.4%) were diagnosed metachronous. The most common site of DM was lung (51.1%), followed by bone (19.1%) and liver (11.5%). Multivariable analysis identified male gender (HR = 1.95, 95% CI 1.23–3.10) hypopharyngeal tumours (HR = 3.28, 95% CI 1.75–6.14), advanced T-stage (HR = 1.61, 95% CI 1.09–2.38), poor differentiation grade (HR = 2.49, 95% CI 1.07–5.78), regional lymph node metastasis (HR = 5.35, 95% CI 3.25–8.79) and extranodal extension of regional lymph nodes metastasis (HR = 3.06, 95% CI 1.39–6.72) as independent prognostic factors for the presence or development of DM. No relation with age was found.

**Conclusion:**

Age is not related to the presence or development of DM. This study emphasizes the importance of screening for DM, especially in males, patients with hypopharyngeal tumours, advanced T-stage, histopathological poor differentiation grade, regional lymph node metastasis and extranodal extension.

## Introduction

In patients with head and neck squamous cell carcinoma (HNSCC), the incidence of distant metastasis (DM) varies between 3 and 52% [1–9]. When patients present with DM, the most affected sites are the lungs, followed by bone and liver [5, 10, 11]. In the last decades, detection of DM improved with the development of imaging techniques (e.g. fluorodeoxyglucose positron emission tomography (FDG-PET) scan), leading to higher detection rates of late DM [3, 12, 13]. The development of DM in HNSCC patients results in an infaust prognosis in most of the cases. Patients with DM receive palliative treatment and unfortunately around 90% decease within 12 months [2]. Patients that are diagnosed with DM prior to treatment are withheld form intensive curative treatment. Nevertheless, approximately 11% patients undergo treatment with curative intent and are shortly afterwards diagnosed with DM [14]. In retrospect, these patients are unnecessarily treated with major consequences for quality of life and healthcare costs [5]. On the other hand, routine screening for DM in all HNSCC patients does not seem rational, because of low incidence of DM in HNSCC patients.

It has been noted that different predictive factors could influence the development of DM, such as advanced T- and N-stage, specific tumour site, poor differentiation grade, extranodal extension and human papillomavirus (HPV) negative oropharyngeal SCC [1, 2, 4, 7, 8, 10, 12, 15, 16]. These predictive factors are globally recognised and adapted in the AJCC/UICC TNM staging system.

Decision making in elderly patients is very complex and decision regret is high in HNSCC patients who receive intensive therapy [17]. Consequently, the question arises whether older age is also a prognostic factor for DM. However, age as a risk factor for DM is less well-studied, and results are contradictory [1, 2, 4, 7–9]. Three studies identified age to be a predictive factor, however it remains unclear whether older [1] or younger [4, 9] patients are at higher risk for developing DM. Therefore, we aimed to identify age and other predictive factors for the development of DM by the analysis of a large cohort of HNSCC patients.

## Patients and methods

### Ethical considerations

The study has been registered in the Research Register of the University Medical Centre Groningen (UMCG). No approval of the Medical Ethical Committee was needed because of the retrospective nature of the study, in accordance with Dutch Medical Research Law legislation.

### Patients

**Fig. 1:**
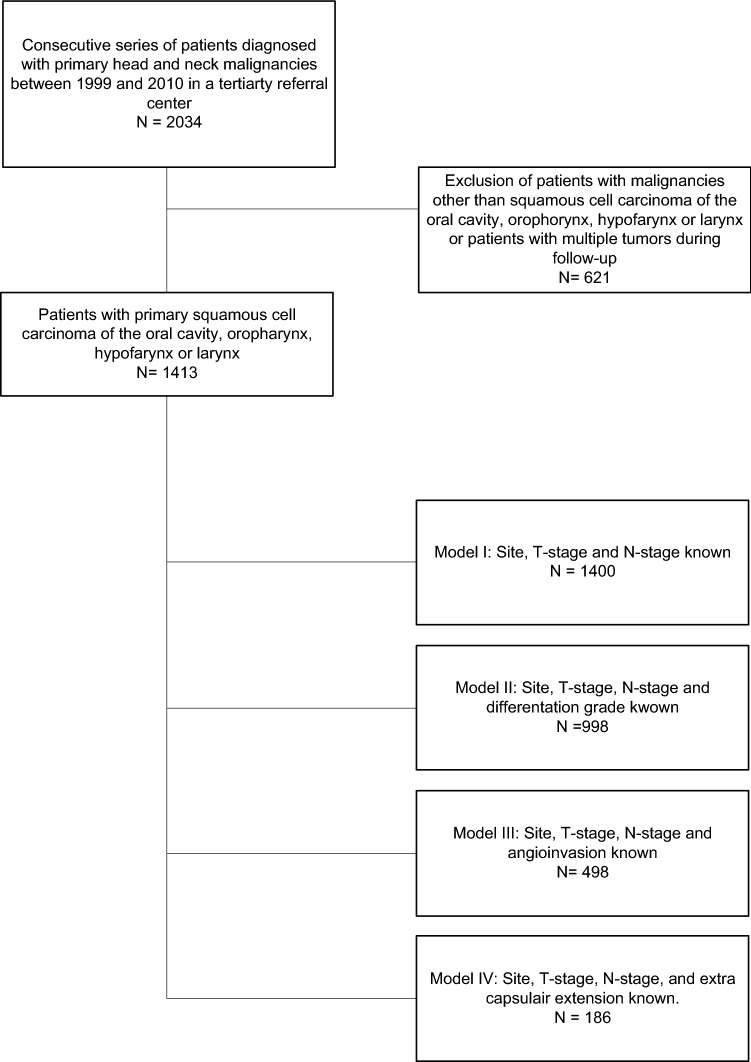
Flow-chart in-/exclusion criteria

This retrospective study includes a cohort of HNSCC patients diagnosed at the UMCG, a tertiary referral head and neck oncology centre in the Netherlands. Data were obtained from the Netherlands Cancer Registry (NCR), managed by the Netherlands Comprehensive Cancer Organization (IKNL). Included patients were 18 years or older, diagnosed with primary oral cavity, oropharyngeal, hypopharyngeal and laryngeal squamous cell carcinoma between 1999 and 2010. Patients with cutaneous squamous cell carcinoma and other tumour types than squamous cell carcinoma were excluded. Patients presenting with multiple or second primary HNSCC were excluded, because of the uncertainty from which site a DM originated. The in- and exclusion of patients for this study are shown in Fig. 1.

Patients with >T1 tumours were standardly screened for regional and intrathoracic metastasis during the primary diagnostic work-up in our hospital by (earlier chest radiography) computed tomography (CT) or magnetic resonance imaging (MRI). Additional MRI, CT, and/or PET were performed in case of clinical suspicion of DM or as part of staging when locoregional failure was diagnosed. Histological confirmation was not standardly performed but only conducted to distinguish between DM and second primary tumours, and was only performed in cases in which further diagnostics had clinical relevance.

### Variables

Data from patient medical files were extracted and included patient characteristics (age, gender, oncological medical history and comorbidities), tumour characteristics (date of diagnosis, tumour site, TNM classification according to the AJCC 7th edition and date of DM) and detailed histopathological information (differentiation grade, perineural growth, angioinvasion and extranodal extension of lymph node metastasis).

Comorbidity was scored using ‘The Adult Comorbidity Evaluation 27’ [18]. Perineural growth, angioinvasion and extranodal extension were only analysed in surgically treated patients, because nonsurgical cases were lacking complete histopathological information.

Depending of the time of discovery of the DM, DM was classified as: simultaneous (discovered with the primary tumour), synchronous (within the first 6 months after diagnosis), and metachronous (after 6 months of initial diagnosis).

### Statistics

Descriptive statistics were used for patient, disease and detailed pathological characteristics. These factors were also stratified by DM during follow-up. To identify potential factors that might be associated with the occurrence of DM, univariable as well as multivariable Cox regression analyses were performed after checking whether the proportional hazards assumption was met by evaluating log minus log plots. For both univariable and multivariable analyses, T-stage was grouped into early stage and advanced stage disease. Hazard ratios (HRs) and 95% confidence intervals (95% CIs) were reported; a 95% CI that did not include 1 or a *p* value below 0.05 was considered statistically significant.

Significant variables in univariable analyses were used for multivariable analysis. Because the multivariable model would include a substantially reduced number of cases when adding all selected variables in one model, four models with subgroups were generated. Model I included all patients whose age, gender, primary tumour site, T- and N-stage were known (*n* = 1399). Model II included patients of which the histological differentiation grade was also known (*n* = 997). Model III included patients of which angioinvasion was also known while differentiation grade was left out (*n* = 473). Model IV also included extranodal extension, while N-stage, differentiation grade and angioinvasion were left out (*n* = 186). Variables included in the final model were selected using a backwards step regression.

For the analysis on the relationship between DM and age, we divided patients in different age categories; <39, 40–49, 50–59, 60–69, 70–79 and ≥80 years.

Kaplan–Meier curves were created to determine patients with and without DM for significant factors according to the multivariable models.

Statistical analyses were performed using IBM SPSS Statistics 23 (IBM, Chicago, IL).

## Results

A total of 1413 patients were included in the study, as shown in Fig. 1. Table 1 shows the patients and disease characteristics. In 131 patients (9.3%) DM developed, of which 27 were diagnosed simultaneously with the primary tumour, 27 were diagnosed synchronous and 77 were diagnosed metachronous. In these cases, the mean time between diagnosis and DM was 20.5 months (SD: 20.8; range 0–116 months, data not shown). Mean age of patients with DM was younger (59.6 years) compared to the patients with no DM (62.6 years). Patients were predominantly male (73.3%). Although the most common primary tumour site was the larynx, most DM originated from the oropharynx (38.9%), followed by the hypopharynx (25.2%), larynx (24.4%) and oral cavity (11.5%). Most primary tumours were diagnosed as a T1 tumour, which had the lowest DM incidence rate (7.6%). Early stage tumours (both T1 and T2) had lower DM incidence rate than advanced stage tumours (both T3 and T4), 5.9 versus 14.7% respectively. Regional lymph node metastasis occurred in 41.1% of all patients. In most patients with DM, also lymph node metastases were detected (77.1%). Of the patients without lymph node metastasis (N0), 3.4% developed DM vs. 17.9% with positive regional lymph nodes. DM was found in 7.1% of well differentiated tumours, while 54.5% originated from tumours with moderate differentiation and 38.4% with poor differentiation. In the DM group, 20.7% of the tumours showed perineural growth, versus 18.3% of the tumours in the group of patients without DM. Angioinvasion was observed in almost one third (30.8%) of the surgically treated patients who developed DM, versus 11.9% without DM. In surgically treated regional lymph nodes (*n* = 221), extranodal extension was more common in the DM group (70.0 versus 46.8%).

**Table 1:** Patient and disease characteristics, overall and stratified by DM at diagnosis or during follow-up

	All (%)	DM (%)	No DM (%)
	1413 (100)	131 (9.3)	1282 (90.7)
Age, mean (SD)	62.3 (11.8)	59.6 (9.0)	62.6 (12.0)
Gender Male Female			
	1036 (73.3) 377 (26.7)	109 (83.2) 22 (16.8)	927 (72.3) 355 (27.7)
Comorbidity (ACE-27) None Mild Moderate Severe Unknown			
	451 (32.0) 412 (29.2) 346 (24.5) 202 (14.3) 2	47 (35.9) 39 (29.8) 29 (22.1) 16 (12.2) 0	404 (31.6) 373 (29.1) 317 (24.8) 186 (14.5) 2
Site Oral cavity Oropharynx Hypopharynx Larynx			
	313 (22.2) 422 (29.9) 137 (9.7) 541 (38.3)	15 (11.5) 51 (38.9) 33 (25.2) 32 (24.4)	298 (23.2) 371 (28.9) 104 (8.1) 509 (39.7)
T-stage T1 T2 T3 T4 Tx Unknown			
	430 (30.6) 428 (30.4) 194 (13.8) 350 (24.9) 5 (0.4) 6	10 (7.6) 41 (31.3) 23 (17.6) 57 (43.5) 0 (0.0) 0	420 (32.9) 387 (30.3) 171 (13.4) 293 (23.0) 5 (0.4) 6
N-stage N0 N1 N2 N3 Unknown			
	843 (59.9) 146 (10.4) 356 (25.3) 63 (4.5) 5	29 (22.3) 14 (10.8) 68 (52.3) 19 (14.6) 1	814 (63.7) 132 (10.3) 288 (22.5) 44 (3.4) 4
M-stage^a^			
M0 M1 Unknown	1380 (98.1) 27 (1.9) 6	104 (79.4) 27 (20.6) 0	1276 (100.0) 0 (0.0) 6
Differentiation grade			
Good Moderate Poor Unknown	195 (19.4) 636 (63.3) 174 (17.3) 408	7 (7.1) 54 (54.5) 38 (38.4) 32	188 (20.8) 582 (64.2) 136 (15.0) 376
Perineural growth^b^			
Yes	75 (18.5) 331 (81.5) 241	6 (20.7) 23 (79.3) 14	69 (18.3) 308 (81.7) 227
No			
Unknown			
Angioinvasion^b^			
Yes No Unknown	51 (13.1) 337 (86.9) 259	8 (30.8) 18 (69.2) 17	43 (11.9) 319 (88.1) 242
Extranodal extension^c^			
Yes No Unknown	95 (50.5) 93 (49.5) 33	21 (70.0) 9 (30.0) 7	74 (46.8) 84 (53.2) 26

^a^M stage at diagnosis of the primary tumor

^b^Only surgically treated cases were included (*n* = 647)

^c^Only cases with surgically treated cervical nodes were included (*n* = 221)

The most common site of DM was lung (51.1%), followed by bone (19.1%) and liver (11.5%) (data not shown).

**Fig. 2:**
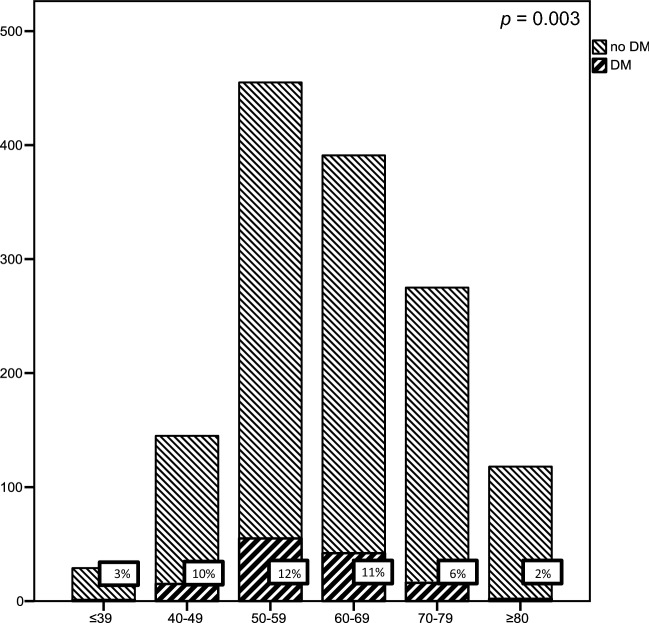
Percentage of DM per age category

For age analysis, categories were applied as described earlier. The number of DM was highest in the 50–59 year old patient population (*n* = 55; 12%), followed by the 60–69 (*n* = 42; 11%), 40–49 (*n* = 15; 10%), 70–79 (*n* = 16; 6%), ≥80 (*n* = 2; 2%) and ≤39 (*n* = 1; 3%) (*p* = 0.003), shown in Fig. 2. Age as either a continuous or categorical variable was not a significant predictor for DM in the multivariable model (Table 3), of which the results of the continuous variable are presented in Table 3.

Table 2 shows the results of the univariable analysis of potential factors influencing the presence or development of DM. Age, male gender, oropharyngeal and hypopharyngeal tumour site, advanced T-stage, positive N status, moderate and poor differentiation grade, the presence of angioinvasion and extranodal extension were identified as significant risk factors associated with DM. Comorbidities, larynx tumours and perineural growth were not identified as significant risk factors for DM.

**Table 2:** Univariable analysis of potential factors related to DM at diagnosis or during follow-up

Variable	HR (95% CI)	*p* value
Age	**0.98** **(0.97–1.00)**	**0.038**
Gender		**0.006**
Male Female	1 **0.53 (0.33–0.83)**	
Comorbidities		0.895
None Mild Moderate Severe	1 0.93 (0.61–1.43) 0.88 (0.55–1.40) 0.82 (0.46–1.45)	
Site		**<0.001**
Oral cavity	1	
Larynx Oropharynx Hypopharynx	1.15 (0.62–2.12) **2.86 (1.61–5.08)** **8.28** **(4.48–15.28)**	0.665 **<0.001** **<0.001**
T-stage^a^		**<0.001**
Early stage	1 **3.85 (2.70–5.48)**	
Advanced stage		
N-stage		**<0.001**
N0	1 **8.18 (5.40–12.39)**	
N+		
Differentiation grade		**<0.001**
Good (Grade I)	1	
Moderate (Grade II) Poor (Grade III)	**2.64 (1.20–5.80)** **7.73 (3.45–17.33)**	**0.016** **<0.001**
Perineural growth^b^		0.400
Yes No	1 1.47 (0.60–3.62)	
Angioinvasion^b^		**0.002**
No Yes	1 **3.80 (1.64–8.77)**	
Extranodal extension^c^		**0.005**
No Yes	1 **3.10 (1.14–6.79)**	

Bold numbers represent significant values (*p* < 0.05)

^a^Early stage represents T1 and T2 tumours, advanced stage represents T3 and T4 tumours

^b^Only surgically treated cases were included (*n* = 647)

^c^Only cases with surgically treated cervical nodes were included (*n* = 221)

Significant factors determined in univariable analysis were tested in a multivariable model, as shown in Table 3. Male gender, hypopharynx tumour site, advanced T-stage, positive N status, poor differentiation grade and extranodal extension were found to be independent predictive factors in multivariable analysis. Male patients had a higher chance to develop DM compared to women, with a hazard ratio (HR) of 1.95, *p* = 0.005. Hypopharyngeal tumours had a higher chance to develop DM with a HR of 3.28, *p* = 0.001. Advanced T-stage was also a significant predictor; with a HR of 1.61, *p* = 0.017. Regional lymph node metastasis was an independent predictor with a HR of 5.35, *p* < 0.001. Poor differentiation grade was an independent predictor of developing DM, with a HR of 2.49 *p* = 0.015. Extranodal extension was an independent predictor with a HR of 3.06, *p* = 0.006. Due to a decrease in included cases in the different models, certain variables such gender, site, and T-stage lost significance in the different models.

**Table 3:**
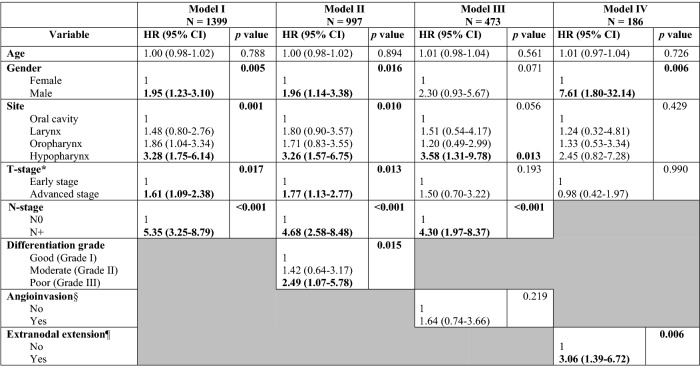
Multivariable analysis of potential factors related to DM at diagnosis or during follow-up

Bold numbers represent significant values (*p* < 0.05). A (multivariable) backwards Cox regression analysis was performed to produce 4 different models to predict distant metastasis, based on statistically significant variables obtained in univariate analysis. Model I included patients with complete information on the variables age, gender, primary tumour site, T- and N-stage (*n* = 1399). Model II included the patients of model I of whom histological differentiation grade was also known (*n* = 997). Model III included patients of model II of whom angioinvasion was also known (*n* = 473). Model IV included patients of model I who also of whom histopathological capsular extension of cervical lymph nodes was also known (*n* = 186)

*Early stage represents T1 and T2 tumours, advanced stage represents T3 and T4 tumours

§Only surgically treated cases were included (*n* = 647)

¶Only cases with surgically treated positive cervical nodes were included (*n* = 221)

**Fig. 3:**
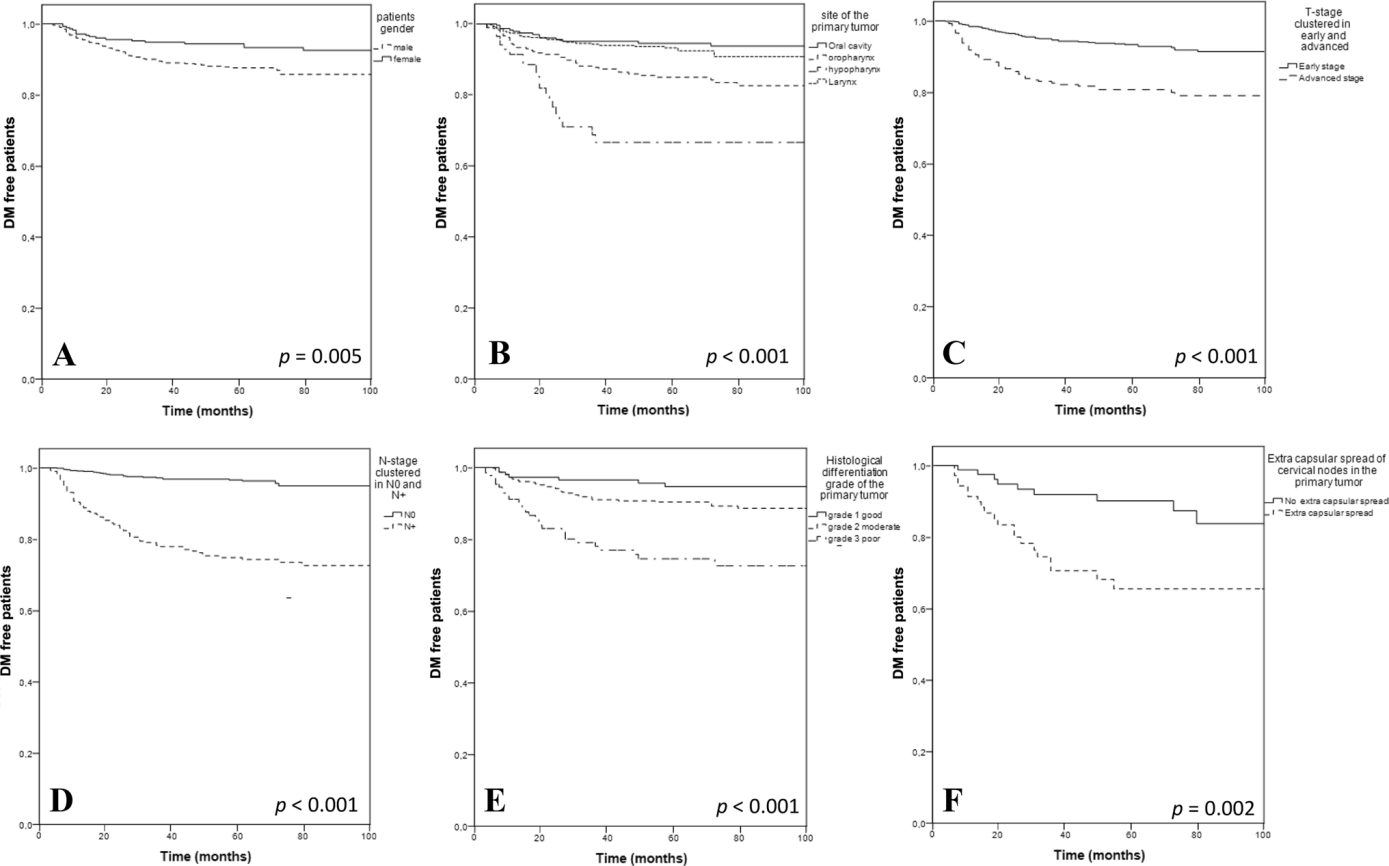
**a**–**f** Kaplan-Meier curves of predictors of DM in HNSCC. Kaplan–Meier curves showing time to metastasis sorted on gender (**a**), the primary tumor site (**b**), T-stage, early: T1-2 vs. advanced: T3-4 (**c**), N-stage, N0 vs. N+ (**d**), histological differentiation grade (**e**) and extra capsular spread in cervical nodes (**f**)

Significant variables in multivariable analysis are also presented in Kaplan–Meier curves in Fig. 3a–f. All variables were also significant in the Kaplan–Meier curves.

## Discussion

In the present study, analysing a consecutive series in one of the largest homogenous published study cohort on this subject, younger age was associated with higher occurrence of DM in univariable analysis, however no relation between age and DM could be observed in multivariable analysis. We confirmed earlier identified independent predictive factors for the development of DM in HNSCC patients: male gender, location of the primary tumour at the hypopharynx, advanced T-stage, regional lymph node metastasis, poor differentiation grade and extranodal extension of regional lymph node metastasis.

In our study, 9% of patients developed DM. However, the reported incidence of DM in HNSCC varies widely in the published literature, between 3 and 52% [1–9]. This is mainly due to different study populations and study designs. The timing of DM diagnosis plays a crucial role in these differences. Some papers study DM at the time of diagnosing the primary tumour, others during complete follow-up and others at autopsy [12]. In our study 20.6% of the DM were discovered at the same time as the diagnosis of the primary tumour.

Age by itself was significantly related to DM in univariable analysis. However, in the four tested models of the multivariable analysis as well as in additional analysis in which patients were divided per age category and related with DM, no significant relation was found between age and DM. In a large study, including over 27,000 patients, older age was identified as an independent significant risk factor for DM, as well as primary site, nodal status, tumour size, and race [1]. However, the authors could not explain these findings and simply state that it is unclear why older patients are at high risk for DM. In contrast, in another large cohort of almost 2,000 HNSCC patients, younger age (<45 years) was found to be significantly associated with the risk of DM [4]. Hypopharyngeal localization, advanced T stage and/or N stage tumour, high histologic grade, and locoregional control were also related to the development of DM. This paper also could not clarify this age related finding. The authors presume that the role of younger age is of limited importance. In a more recently published study, also younger age at diagnosis was discovered a risk factor for developing pleural metastasis [9]. Likewise in this study, the authors do not give an explanation for this finding, concluding none of the above mentioned studies can interpret their age-related findings. In concordance with our results, few other studies also didn’t find an association between age and DM [7, 8]. A retrospective study analysing 130 advanced stage HNSCC identified clinically palpable neck disease (N1‐3), histological evidence of metastatic nodal disease, extranodal extension, and three or more positive lymph nodes as predictors for developing DM [7]. Age, gender, primary site, history of radiation therapy, perineural invasion and tumour grade were not associated with a higher risk for DM. In another study, development of DM was not related with age, while N stage, T stage, and pre-treatment maximum standardized uptake value of the lymph node were strongly associated [8]. In a larger study including over 1,200 HNSCC patients, age was also not related to the frequency of DM [2]. None of these studies give an explanation for their age related findings. Discrepancies in age related findings between this study and the above mentioned studies could partly be explained by differences in study design. Some studies include a cross-sectional study design, measuring DM only at diagnosis, while others performed a longitudinal study including both DM found at diagnosis as well as during follow-up after treatment. This explains the differences in reported incidence of DM, which might also impact other results, such as age-related findings. Furthermore, the method of detection of DM is different among the above discussed studies. In most studies, description of the diagnostic process (like for example the applied imaging modalities) is lacking. Even within a study, work-up might vary due to differences in work-up between high-risk and lower-risk patients, which can also affect results. Differences in the epidemiological profile of patients among studies may also play a role. Our study population contains all HNSCC in a between 1999 and 2010, while others included only advanced staged HNSCC patient, for instance specifically patients that underwent intensity-modulated radiotherapy or only patients with pleural metastasis. All these factors might have consequences for age analysis. Finally, limited sample size and thus the low power of many currently available studies may also partly explain conflicting results. However, also studies with large power found opposite results. All in all, the explanation for these different results concerning age and DM remains unclear.

In a recently published study, CART analysis (classification and regression tree) was used to assess the impact of age on the survival of patients with HNSCC. Age was a significant prognostic factor in predicting 5‐year disease‐specific survival, based on the uni‐ and multivariate analyses. However, in their CART model, the authors found that age plays only a minor role in HNSCC survival. This method revealed that the impact of age varied for different patient groups according to the presence or absence of other prognosticators. This was different to our results; however, comparison between these studies is difficult as the cited study did not specifically investigated DM [19].

We identified the presence of regional lymph node metastasis as the strongest independent predictor for DM. The important role of advanced N-stage as a predictor of DM development has already been described in numerous studies and is now also supported by our findings [1, 4, 7, 8, 10, 12, 20]. Two of these studies also found extranodal extension an independent predictor of DM, in concordance with our results [7, 21]. While in one other study, extranodal extension was not a significant predictive factor for DM [22].

Hypopharyngeal tumours seem to give the highest risk of DM [1, 2, 4, 10, 20, 23]. In line with these findings, in our cohort patients with hypopharyngeal tumours had more than three times as high chance to develop DM compared to their counterparts with oral cancer.

In this study, advanced tumour stage and poor differentiation grade were also found to be a significant independent predictor of DM. These factors were also described as high risk factors for DM in a review on DM in HNSCC [12].

Male gender was also found to be an independent predictor of the development of DM. Others also investigated the relation between gender and the development of DM, most of these studies didn’t find male gender to be a significant predictive factor [1, 2, 5, 8, 9]. In other tumours this trend was also observed, for instance in malignant melanoma [24]; however, the explanation for this finding in HNSCC remains unclear.

Also the effect of angioinvasion and perineural growth on the development of DM was studied. Interestingly, neither angioinvasion nor perineural growth were significant independent predictors for DM, which is confirmed by two other studies [7, 10]. This finding is remarkable, as perineural growth is considered to be a robust prognostic factor in cancer. However, it has found to be associated with an increased risk of local recurrence and regional metastasis rather than with DM [25, 26].

Overall, the most frequent locations for DM in the present study were the lungs (51.1%), followed by the bones (19.1%) and the liver (11.5%). In line with our findings, this pattern is also described in other studies [5, 10, 11]. In a large study, in which 832 patients with HNSCC were autopsied, most metastasis were found in the lungs (80%), followed by mediastinal nodes (34%), liver (31%) and bone (31%), indicating that a clinical diagnosis of DM might be less precise than a pathological diagnosis [23]. Because of the very poor prognosis of patients with DM, in case of clinical suspicion, further diagnostics are often omitted as it has no clinical relevance.

In this study, DM only occurred in the first 120 months of follow-up. Typical Kaplan–Meier curves of DM in HNSCC show a rapid increase between months 0 and 8, a slow increase between months 8 and 24, and a plateau between months 24 and 84.5 indicating the absence of late distant metastasis [10].

This study included a very large series of consecutive cases and well-documented records. Because the retrospective nature of this cohort study, missing data were expected due to incomplete case records. However, our study population was large and adequate to perform multivariable analyses. Despite, some risk factors described in literature (e.g. lymph node size and bilateral and low neck level involvement) [2, 21, 22, 27, 28] could not be included in our analysis due to high number of missing data in the retrospective database. In addition, we did not have information on HPV status of the oropharyngeal tumours. Though, this would be interesting to analyse, as HPV-related oropharyngeal cancer is known to be associated with higher stage at diagnosis and more favourable outcomes [16].

Due to the improved locoregional control and the increasing number of HNSCC survivors in the last decades, data on DM development gained more importance, recently [12]. On the other hand, recognizing patients with high chance of developing DM is essential, as these patients need to be screened before they undergo intensive treatment while costly imaging modalities can be spared in their counterparts who do not likely develop DM. According to the present study, regional lymph node metastasis is the strongest predictor for DM in HNSCC patients. However, more factors should be considered as indicators for screening, such as male gender, hypopharyngeal tumours, advanced T-stage, poor differentiation grade, regional lymph node metastasis and extranodal extension. Based on our study, a large, representative and homogenous cohort, age does not play a role in the development of DM. Therefore, in all patients with high risk of DM development, extensive screening should be considered, irrespective of patients’ age. Screening should include the whole body, as metastasis may occur in the lungs, but also in the liver, the bones or in multiple organs. For this reason, the most appropriate method seems to be FDG-PET/CT. However, the method of screening was not the subject of this study.
